# Mechanisms Underlying the Synergistic Action of Insulin and Growth Hormone on IGF-I and -II Expression in Grass Carp Hepatocytes

**DOI:** 10.3389/fendo.2018.00336

**Published:** 2018-06-21

**Authors:** Quan Jiang, Jin Bai, Mulan He, Karen W. Y. Yuen, Anderson O. L. Wong

**Affiliations:** ^1^School of Biological Sciences, The University of Hong Kong, Hong Kong, China; ^2^College of Life Sciences, Sichuan University, Chengdu, China

**Keywords:** insulin, growth hormone, insulin-like growth factor, insulin receptor, growth hormone receptor, signal transduction, hepatocytes, grass carp

## Abstract

In mammals, insulin is known to modify growth hormone (GH)-induced IGF-I expression at the hepatic level, which also contributes to the functional crosstalk between energy homeostasis and somatotropic axis. However, the studies on the comparative aspects of this phenomenon are limited and the mechanisms involved have not been fully characterized. Using a serum-free culture of grass carp hepatoctyes, the functional interaction between GH and insulin on hepatic expression of IGF-I and -II was examined in a fish model. In carp hepatocytes, GH could up-regulate IGF-I and -II mRNA expression via the JAK_2_/STAT_5_, MEK/ERK and PI3K/Akt pathways. These stimulatory effects were mimicked by insulin via activation of the PI3K/Akt but not MEK/ERK and P38 MAPK cascades. Although insulin did not activate JAK_2_ and STAT_5_ at hepatocyte level, insulin-induced IGF-I and -II mRNA expression were highly dependent on the normal functioning of JAK_2_/STAT_5_ pathway. In parallel experiments, insulin co-treatment was found to markedly enhance IGF-I and -II responses induced by GH and these potentiating effects were mediated by insulin receptor (InsR) but not IGF-I receptor. Interestingly, co-treatment with GH also enhanced insulin-induced InsR phosphorylation with a current elevation in protein:protein interaction between GH receptor and phosphorylated InsR and these stimulatory effects were noted with further enhancement in STAT_5_, ERK_1/2_ and Akt phosphorylation at hepatocyte level. Consistent with these findings, the potentiating effects of GH and insulin co-treatment on IGF-I and -II mRNA expression were found to be suppressed/abolished by inhibiting JAK_2_/STAT_5_, MEK/ERK and PI3K/Akt but not P38 MAPK pathways. These results, as a whole, suggest that insulin and GH can act in a synergistic manner in the carp liver to up-regulate IGF-I and -II expression through protein:protein interaction at the receptor level followed by potentiation in post-receptor signaling.

## Introduction

Growth hormone (GH) secreted from the pituitary is essential for body growth, metabolism and tissue maintenance/repairing. These biological effects are mediated mainly by insulin-like growth factor (IGF) produced at the hepatic level, which forms the basis of the “somatomedin hypothesis” for GH action (1). In mammals, two forms of IGF, IGF-I and IGF-II, have been identified with IGF-I as the dominant form expressed in the liver. IGF-I plays a key role in postnatal growth mediated by somatotropic axis (2) and is highly responsive to GH induction via GH receptor (GHR) coupled to JAK_2_/STAT_5_, MEK/ERK and PI3K/Akt pathways (3). In contrast, IGF-II is mainly involved in placental growth and prenatal development of the fetus and its expression in general is not responsive to GH (4, 5). In fish models, IGF-I and -II are readily detectable at the tissue level, especially in the liver (6), and unlike mammals, IGF-I and -II production/secretion at the hepatic level can be up-regulated by GH treatment, e.g., in tilapia (7) and salmon (8), which lend support to the idea that the two IGFs are both involved in the somatotropic actions of GH in fish species (9). Besides IGF-I and -II, a new form of IGF, namely IGF-III, has also been reported in fish models and its expression is largely restricted to the gonad, e.g., in zebrafish, tilapia and medaka (10). However, gonadal expression of IGF-III is not affected by GH treatment, e.g., in tilapia (11), and its functional role in GH action/signaling is questionable.

In mammals, the portal vascular link between pancreatic islets and the liver provides the anatomical basis for direct modulation of hepatic functions by pancreatic hormones, including insulin and glucagon (12). At the hepatic level, GH is known to interact with insulin to regulate carbohydrate and lipid metabolism (13). Meanwhile, the sensitivity of hepatocytes for GH-induced IGF-I expression can also be modified by insulin. For examples, insulin deficiency (e.g., type I diabetes) can lead to parallel drop in GHR expression and reduced levels of IGF-I transcript in the liver (14) and these inhibitory effects can be reverted by insulin replacement (15). Under chronic hyperinsulinema (e.g., obesity and type II diabetes) or prolonged treatment with insulin, GH resistance at the hepatic level is commonly observed, which can be attributed to the inhibitory effects of insulin on JAK_2_/STATs signaling (16, 17) and GHR gene transcription via the MEK/ERK and PI3K/Akt pathways (18, 19). In rat hepatocytes, short-term treatment with insulin can stimulate IGF-I expression by increasing its transcript stability but with no notable effect on gene transcription (20). In the same model, insulin either has no effect (21) or potentiates GH-induced IGF-I mRNA expression (22). For the case with potentiating effect, the mechanisms involved are still unclear but suspected to be mediated through a crosstalk between GH and insulin at the level of post-receptor signaling (23). This idea is supported by the report in H4IIE hepatoma, in which insulin co-treatment can enhance GH-induced MEK_1/2_ and ERK_1/2_ phosphorylation independent of JAK_2_/STAT_5_ activation (24). Of note, the involvement of MEK/ERK pathway in the potentiating effect of insulin on IGF-I expression induced by GH has yet to be confirmed.

Insulin potentiation of GH-induced IGF-I expression has also been reported in hepatocytes from chicken (25). However, the results based on similar studies in fish species are variable and different from that of mammals. In salmon hepatocytes, insulin has no effect on basal but inhibits GH-induced IGF-I mRNA expression (26). In the same model, similar treatment with insulin can elevate basal and potentiate GH-induced IGF-II gene expression (8). Interestingly, in tilapia hepatocytes, insulin can exert opposite effects on the two IGFs, with inhibition on IGF-I but stimulation on IGF-II mRNA expression. Similar to salmon hepatocytes, insulin can also enhance GH-induced IGF-II expression but with no effect on the corresponding response for IGF-I (7). Apparently, insulin potentiation of GH-induced IGF-II expression is a common phenomenon in fish models whereas the corresponding effects on IGF-I are species-specific. Given that the comparative studies for the effects of insulin on GH-induced IGF expression are still limited in lower vertebrates and the mechanisms involved, e.g., for the potentiating effect of insulin on IGF-II expression, are largely unknown, *in vitro* studies were conducted in serum-free culture of grass carp hepatocytes to unveil the functional interaction between insulin and GH on IGF-I and -II regulation in the carp liver. Grass carp was used as the animal model as it is a representative of Cyprinids and has a high market value in Asian countries including China. In this study, we sought to address the questions on (i) What are the effects and underlying mechanisms for GH and insulin on IGF-I and -II expression in the carp liver? (ii) Do GH and insulin interact at hepatocyte level to modify IGF-I and -II expression? and (iii) What are the cellular mechanisms involved in the functional interaction between GH and insulin for IGF-I and -II regulation? Using a pharmacological approach coupled to direct probing of activation status for selected kinases involved in the post-receptor signaling, the signal transduction for hepatic expression of the two IGFs induced by GH and insulin were elucidated in grass carp hepatocytes. The functional role of insulin receptor (InsR) and IGF-I receptor (IGF1R) in IGF -I and -II regulation by GH and insulin co-treatment as well as the post-receptor signaling responsible for GH and insulin interaction were also examined. Our studies for the first time provide evidence that GH and insulin can act in a synergistic manner at the hepatocyte level to enhance IGF-I and -II expression in a fish model through protein:protein interaction at receptor level with subsequent potentiation in post-receptor signaling.

## Materials and methods

### Animals and test substances

One-year-old grass carp (*Ctenopharyngodon idellus*) with body weight of 1.8–2.1 kg were obtained from local wholesale market and maintained in well-aerated aquaria at 20°C under 12-h light:12-h dark photoperiod for at least 2 weeks prior to experimentation. Since the fish at this stage were sexually immature and sexual dimorphism was not apparent, grass carp of mixed sexes were used for hepatocyte preparation according to the protocol approved by the Committee for Animal Use in Teaching and Research at the University of Hong Kong (Hong Kong). Porcine GH and human insulin were obtained from Sigma-Aldrich (St. Louis, MO) while the blockers for IGF1R/InsR, including picropodophyllin (PPP) and hydroxy-2-naphthalenyl-methyl phosphonic acid (HNMPA), and inhibitors for target pathways, including 2-cyano- 3-(3,4-dihydroxy-phenyl)-N-(benzyl)- 2-propenamide, 2-cyano-3-(3,4-dihydroxyphenyl)-N-(phenylmethyl)-2-propenamide (AG470), N′-((4-oxo-4H-chromen- 3-yl) methylene) nicotinohydrazide (NICO), 2-(2-amino-3-methoxyphenyl)4H- 1-benzopyranone (PD98059), 5-(2-phenylpyrazolo [1,5-a]pyridin-3-yl)-1H-pyrazolo- [3,4-c]pyridazin-3-amine (FR180204), 4-[5-(4-fluorophenyl)-2- [4-(methyl-sulfonyl)-phenyl]-1H-imidazolyl] -pyridine (SB203580), 2-(4-morpholinyl)-8-phenyl-4H-1- benxopyrin-4- one (LY294002), 1L6-hydroxymethylchiro-inositol-2-*O*-methyl-3-*O*-octadecyl-glycerocarbonate (HIMOC) and rapamycin, were acquired from Calbiochem (San Diego, CA). Stock solutions of test substances, except for GH and insulin which were dissolved in PBS, were prepared in DMSO and stored frozen at −80°C in small aliquots. On the day of experiment, frozen stocks of test substances were thawed on ice and diluted with culture medium to appropriate concentrations 10 min prior to their administration to hepatocyte culture. The final dilutions of DMSO were always maintained at ≤ 0.1% and did not affect IGF-I and -II gene expression.

### Primary culture of grass carp hepatocytes

Primary culture of grass carp hepatocytes was prepared by collagenase digestion method as described previously (27). Briefly, liver slices (~1.0 g per fish) freshly excised from 5–6 grass carp were rinsed for 2–3 times to remove blood clots and diced into small fragments of 0.5 mm in thickness using a McIlwain Tissue Chopper (Cavey Lab, Guildford Surrey, UK). Fragments prepared were then incubated with type IV collagenase (500 U/ml) and DNase II (0.01 mg/ml) at 28°C for 30 min. After enzyme digestion, the fragments were dispersed by trituration, filtered through sterilized nylon mesh (~30 μm pore size), and seeded in PEI-precoated 24-well plates at ~0.7 × 10^6^ cells/ml/well in DMEM/F12 medium (pH 7.6) without serum supplement. A serum-free culture was used to avoid the confounding effects of GH and insulin present in serum supplement. Hepatocytes prepared with a viability of 92–97% were cultured overnight at 28°C under 5% CO_2_ and saturated humidity to allow for the recovery from enzyme digestion. On the next day, static incubation with test substances was performed at the dose and duration as indicated for individual experiments.

### Real-time PCR for IGF-I and -II mRNA measurement

After drug treatment, total RNA was isolated from hepatocytes using TRIzol (Thermo Fisher), reversely transcribed with Superscript II (Invitrogen), and subjected to real-time PCR for IGF-I and -II mRNA measurement using a LightCycler^®;^ 480 SYBR Green I Master Kit (Roche, Basel, Switzerland) with a Rotor Gene-Q qPCR System (Qiagen). For real-time PCR targeting grass carp IGF-I (Accession no GU583648.1) and IGF-II (Accession no FJ410929.1), gene-specific primers for carp IGF-I (TTCAAGTGTACCATGCGCTG and ACCGTCTTGAATTAGGCCCA) and IGF-II (AGACCCTTTGCGGTGGAGA and GGAAACATCTCGCTCGGACT) were used for quantitative PCR for respective gene targets. PCR reactions for the two IGFs were conducted for 35 cycles with denaturation at 95°C for 30 s, annealing at 62°C for 30 s, and extension at 72°C for 30 s and PCR signals for IGF-I and -II mRNA were recorded by the end of individual cycles with acquiring temperature at 88°C for 20 s. After real-time PCR, the authenticity of PCR products were routinely confirmed by melting curve analysis. In this case, the PCR products obtained were found to be 201 bp in size with *Tm* value at 93°C for IGF-I and 182 bp in size with *Tm* value at 90°C for IGF-II, respectively. Serial dilutions of plasmid DNA carrying the coding sequences for grass carp IGF-I and -II were used as the standards for data calibration and parallel measurement of 18S RNA expression was used as the internal control as described previously (27).

### RT-PCR of InsR and IGF1R expression

To examine InsR and IGR1R expression in the carp liver, RT-PCR was conducted in total RNA isolated from carp hepatocytes with DNase I digestion followed by reverse transcription in the presence (as “+RT”) or absence of Superscript II (as “-RT”). The RT samples prepared were used as the templates for PCR using specific primers for grass carp InsRa (Accession no KP713801.1; ACTTTCTCCTGTTCCGTGTC and GTAGTTGTCCTCCACCGAGT), InsRb (Accession no KR866114.1; GTCCACCACCAACCCTGAA and TCCCGCCCTTGCGATAAT) and IGF1R (Accession no EU816193.1; AACCAGGGTGGCCATCAAAA and GAGGTGTTCTCAGCGGATCG), respectively. PCR reactions were performed for 38 cycles with denaturation at 95°C for 30 s, annealing at 58°C for 30 s and extension at 72°C for 30 s. PCR products obtained were resolved in 1% agarose gel and visualized by ethidium bromide staining. Parallel RT-PCR for InsR and IGF1R were also performed with RT sample prepared from the carp pituitary to serve as a positive control. In this experiment, RT-PCR for β actin (27) was also performed to serve as the internal control.

### Western blot for kinase activation in carp hepatocytes

To study the effect of drug treatment on kinase activation at the hepatic level, Western blot was conducted in carp hepatocytes as described previously (28). After treatment with insulin (with/without GH co-treatment), carp hepatocytes were lysed using RIPA buffer supplemented with a complete cocktail of protease and phosphatase inhibitors (Roche). The lysate prepared was then size-fractionated by SDS-PAGE, transblotted onto a nitrocellulose membrane, and subjected to Western blot using the antibodies targeting the phosphorylated form (“P-” form) and total protein (“T-” form) of JAK_2_ (1:1,000), STAT_5_ (1:1,000), MEK_1/2_ (1:1,000), ERK_1/2_ (1:5,000), P38 MAPK (1: 1,000) and Akt (1:1,000) (Cell Signaling Technology), respectively. To test the effect of GH on InsR activation by insulin in carp hepatocytes, Western blot was also conducted with antibodies for the phosphorylated form (1:2,000, Abcam) and total protein of InsR (1: 2,000, Santa Cruz). In these studies, parallel blotting of β actin was used as the loading control. Although the antibodies used in our study were raised against the respective targets in human or mouse model, the epitopes detected are highly conserved in fish counterparts and the antibodies adopted in our study have been used previously in probing the respective targets in grass carp (27), goldfish (29) and rainbow trout (30).

### Co-immunoprecipitation of GHR and InsR in carp hepatocytes

To evaluate the possible interaction of GHR and InsR in the carp liver with GH and insulin co-treatment, co-immunoprecipitation experiment was performed in carp hepatocytes. In this case, hepatocytes were cultured at 15 × 10^6^ cells/8 ml/100 mm dish and challenged for 15 min with either GH or insulin alone or with GH and insulin co-treatment. After treatment, protein samples were prepared using a Mem-PER Membrane Protein Extraction Kit (Thermo Fisher). A small aliquot of individual samples was reserved as the input control for Western blot (as “input” sample) and the rest of the samples were subjected to immunoprecipitation with a Pierce Co-immunoprecipitation Kit (Thermo Fisher) using an antiserum raised against the N-terminal of carp GHR (1: 2,000) for target pull-down. Parallel immunoprecipitation was also conducted with mouse IgG to serve as the negative control. After sample loading onto protein A agarose with GHR antiserum, the washing steps and protein elution were conducted according to the instructions of the kit manual. The eluate (as “pull-down” sample) was used as the samples for Western blot with antibodies for phosphorylated form and total protein of InsR as described in preceding section. Regarding the input control, Western blot with InsR antibodies and GHR antiserum were also performed in the input samples. In this experiment, parallel blotting for β actin was routinely used as the loading control.

### Data transformation and statistical analysis

For real-time PCR of IGF-I and -II mRNA, standard curves constructed with the respective plasmid DNAs with a dynamic range of ≥10^5^, amplification efficiency ≥ 98% and correlation coefficient ≥ 0.95 were used for data calibration under unsupervised mode with RotorGene Q-Rex software (Qiagen). Given that expression levels of 18S RNA in carp hepatocytes did not exhibit significant changes with drug treatment in our studies, the raw data for IGF-I and -II mRNA (in femtomole target detected per 10^6^ cells) were transformed as a percentage of the mean value in the control group without drug treatment (as “%Ctrl”). Data presented (Mean ± SEM) are pooled results from four to six separate experiments (*N* = 4–6) and were analyzed with one-way (for dose dependence/interaction studies) or two-way ANOVA (for time course) followed by Tukey *post-hoc* test. Differences between treatment groups were considered as significant at *P* < 0.05.

## Results

### Regulation of IGF gene expression by GH and insulin in carp hepatocytes

**Figure 1:**
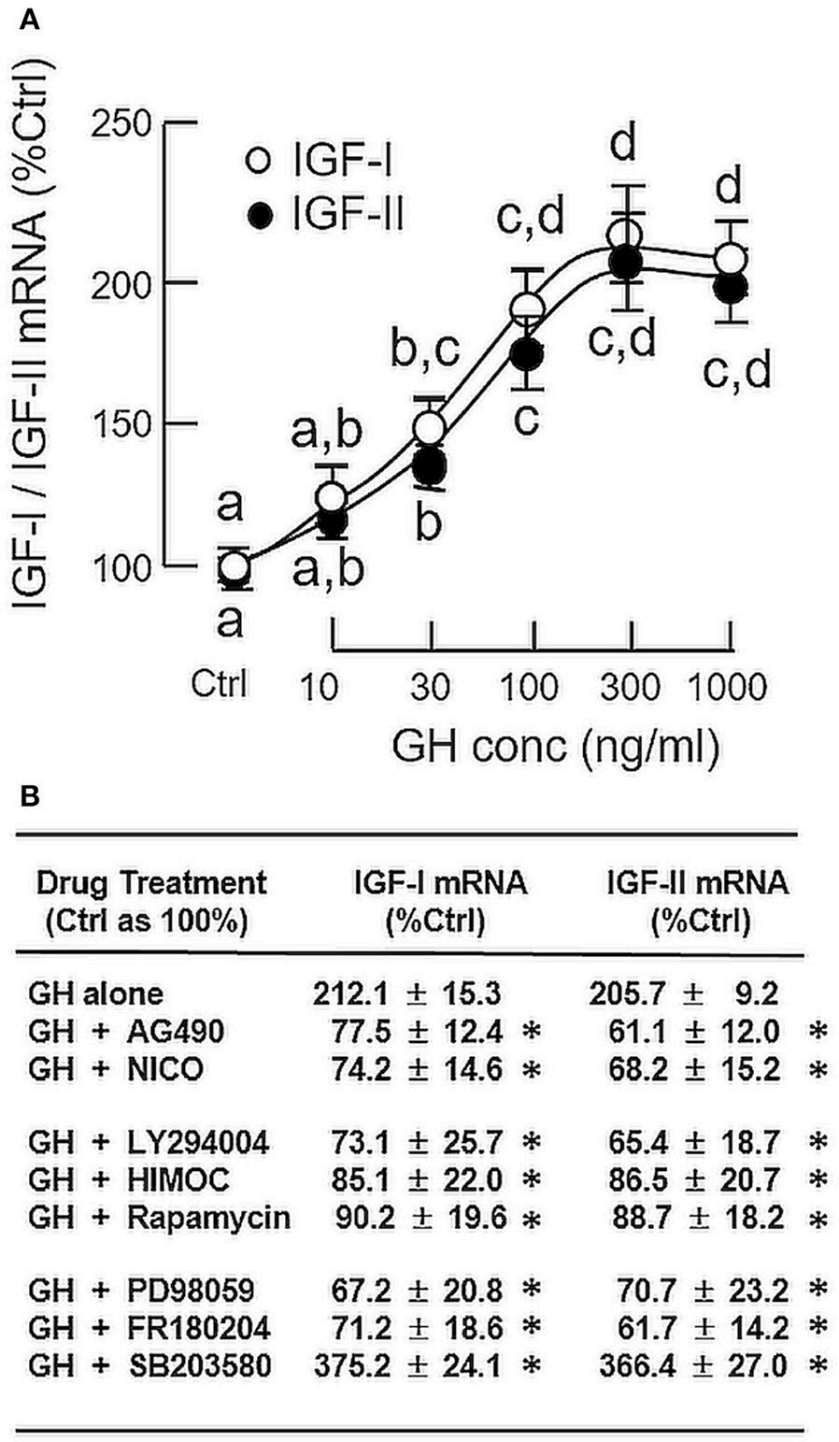
Regulation of IGF-I and -II expression by GH in grass carp hepatocytes. **(A)** Effects of increasing doses of GH treatment (10–1,000 ng/ml) on IGF-I and -II mRNA expression. **(B)** JAK_2_/STAT_5_, MAPK, and PI3K/Akt pathways in IGF-I and -II mRNA expression induced by GH. For dose dependence experiment, static incubation of hepatocytes was conducted for 24 h with increasing levels of GH as indicated. For parallel experiments to examine the signal transduction involved in GH actions, hepatocytes were treated for 24 h with GH (300 ng/ml) in the presence of the JAK_2_ inhibitor AG490 (20 μM), STAT_5_ inhibitor NICO (20 μM), PI3K inhibitor LY294004 (10 μM), Akt inhibitor HIMOC (10 μM), mTOR inhibitor rapamycin (20 nM), MEK_1/2_ inhibitor PD98059 (10 μM), ERK_1/2_ inhibitor FR180204 (2 μM), and P38 MAPK inhibitor SB203580 (5 μM), respectively. After treatment, total RNA was extracted from the cell culture and subjected to real-time PCR with primers specific for grass carp IGF-I and -II, respectively. Parallel real-time PCR for 18S RNA was also conducted to serve as the internal control. For IGF data presented (Mean ± SEM), the groups denoted by different letters (for dose dependence) or with asterisks (for signal transduction) represent a significant difference at *P* < 0.05.

To confirm that grass carp hepatocytes could still retain its responsiveness to GH under a serum-free condition, static incubation with increasing concentrations of GH (10–1000 ng/ml) was performed for 24 h without serum supplementation. During the process, the cell culture did not exhibit noticeable changes in overall morphology. As shown in Figure 1A, GH treatment was effective in increasing IGF-I and -II mRNA levels in a dose-dependent manner with the maximal responses occurred in the 300–1000 ng/ml dose range. Based on these findings, the duration of drug treatment for subsequent studies, unless stated otherwise, was fixed at 24 h under a serum-free culture condition. In mammals, the biological actions of GH are mediated through GHR coupled with the JAK_2_/STAT_5_, MAPK, and PI3K/Akt pathways (3). To shed light on the signal transduction for GH-induced IGF-I and -II expression in the carp liver, the serum-free culture of carp hepatocytes was challenged with GH (300 ng/ml) in the presence of the pharmacological inhibitors targeting different components of the JAK_2_/STAT_5_, MAPK, and PI3K/Akt cascades (Figure 1B). In this case, IGF-I and -II mRNA expression induced by GH were found to be totally abolished by co-treatment with the JAK_2_ inhibitor AG490 (20 μM), STAT_5_ blocker NICO (20 μM), PI3K inactivator LY294004 (10 μM), Akt inhibitor HIMOC (10 μM), mTOR inhibitor rapamycin (20 nM), MEK_1/2_ blocker PD98059 (10 μM) and ERK_1/2_ inhibitor FR180204 (2 μM), respectively. However, the opposite was true with treatment of the P38 MAPK inhibitor SB203580. In carp hepatocytes, SB203580 (5 μM) not only could elevate basal (data not shown) but also enhance the stimulatory effects of GH on IGF-I and -II gene expression.

**Figure 2:**
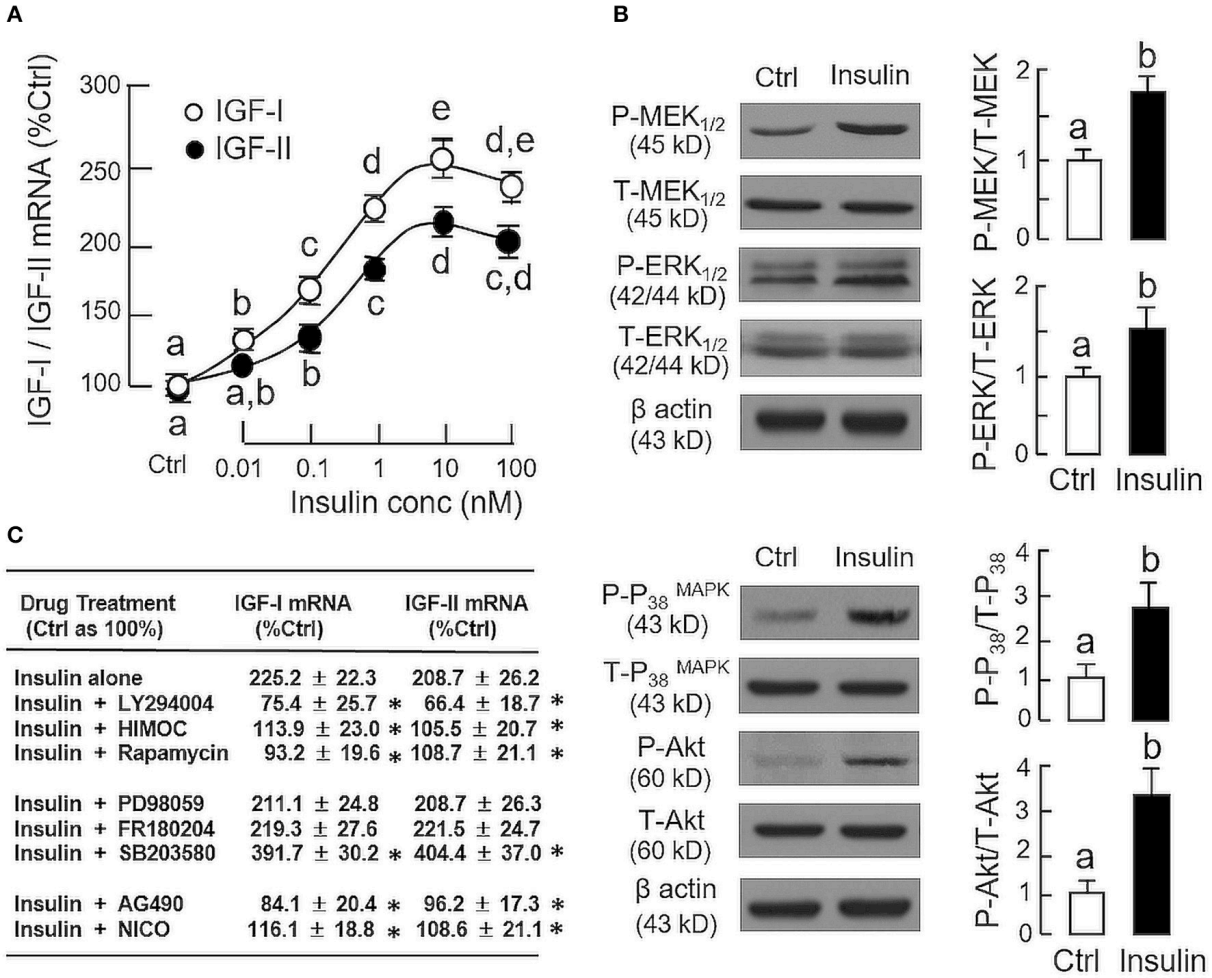
Regulation of IGF-I and -II expression by insulin in grass carp hepatocytes. **(A)** Effects of increasing doses of insulin treatment (0.01–100 nM) on IGF-I and -II mRNA expression. **(B)** Insulin stimulation on protein phosphorylation of MEK_1/2_, ERK_1/2_, P38 MAPK, and Akt in carp hepatocytes. **(C)** PI3K/Akt, MAPK, and JAK_2_/STAT_5_ pathways in IGF-I and -II mRNA expression induced by insulin. For dose dependence study, hepatocytes were challenged for 24 h with increasing levels of GH as indicated. For parallel experiments to unveil the signal transduction for insulin actions, hepatocytes were treated for 24 h with insulin (10 nM) in the presence of the PI3K inhibitor LY294004 (10 μM), Akt inhibitor HIMOC (10 μM), mTOR inhibitor rapamycin (20 nM), MEK_1/2_ inhibitor PD98059 (10 μM), ERK_1/2_ inhibitor FR180204 (2 μM), P38 MAPK inhibitor SB203580 (5 μM), JAK_2_ inhibitor AG490 (20 μM), and STAT_5_ inhibitor NICO (20 μM), respectively. For protein phosphorylation, cell lysate was prepared from carp hepatocytes after 15-min treatment with insulin (10 nM) and subjected to Western blot using specific antibodies for the phosphorylated form (*P*-form) and total protein (*T*-form) of MEK_1/2_, ERK_1/2_, P38 MAPK, and Akt, respectively. The signals for the two forms of target proteins were then quantitated by Image J and expressed as a ratio of P-form/T-form in the bar graphs on the right. In these experiments, parallel blotting of β actin was used as the loading control. For the data presented, the groups denoted by different letters or asterisks represent a significant difference at *P* < 0.05.

In parallel study to test the effect of insulin on IGF expression at the hepatic level, increasing doses of insulin (0.01–100 nM, 24 h), similar to GH, could elevate IGF-I and -II mRNA levels in carp hepatocytes in a concentration-related fashion with maximal responses observed in the 10–100 nM dose range (Figure 2A). Given that insulin actions are well-documented to be mediated by MAPK and PI3K/Akt cascades (31), the possible involvement of these signaling pathways in insulin-induced IGF-I and -II expression was also examined. In carp hepatocytes, a 15-min treatment with insulin (10 nM) was effective in triggering rapid phosphorylation of MEK_1/2_, ERK_1/2_, P38 MAPK and Akt (Figure 2B). However, similar treatment was found to have no effect on JAK_2_ and STAT_5_ phosphorylation (Supplemental Figure 1). To test the possible role of MAPK and PI3K/Akt pathways in IGF regulation by insulin, insulin-induced IGF-I and -II expression were examined with co-treatment of pharmacological inhibitors for the respective cascades. As shown in Figure 2C, up-regulation of IGF-I and -II mRNA levels induced by insulin (10 nM) could be negated by co-treatment with the PI3K inhibitor LY294004 (10 μM), Akt inactivtor HIMOC (10 μM) and mTOR blocker rapamycin (20 nM), respectively. Parallel co-treatment with the MEK_1/2_ inhibitor PD98059 (50 μM) or ERK_1/2_ inhibitor FR180204 (2 μM) was not effective in these regards. Similar to the study with GH induction, the P38 MAPK inhibitor SB203580 (5 μM) could also enhance the stimulatory effects of insulin on IGF-I and -II transcript expression. Of note, although insulin did not alter JAK_2_ and STAT_5_ phosphorylation in carp hepatocytes, the JAK_2_ inhibitor AG490 (50 μM) and STAT_5_ blocker NICO (50 μM) were effective in blocking insulin-induced IGF-I and -II gene expression.

### Functional interaction of GH and insulin on IGF-I and -II expression

**Figure 3:**
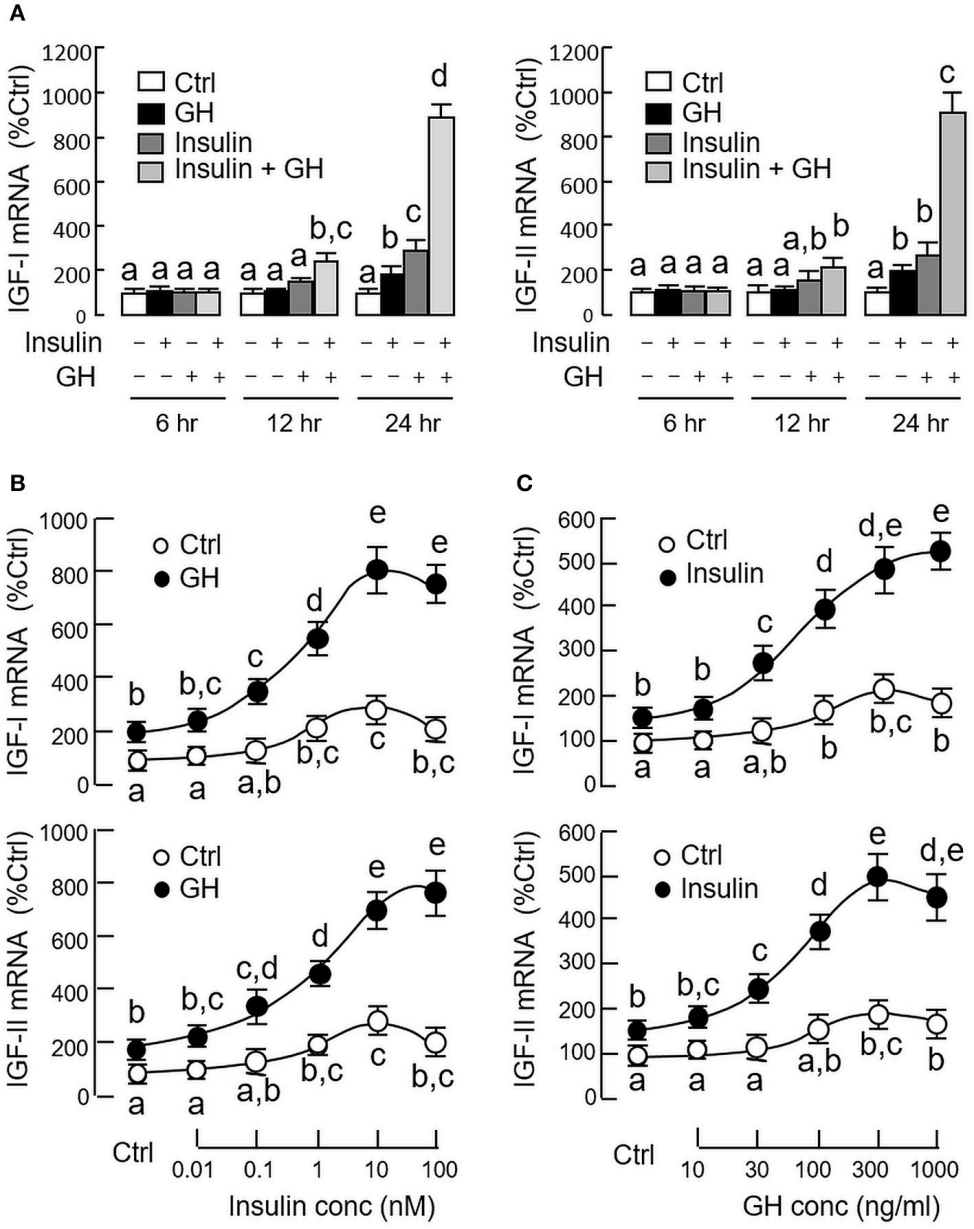
Synergistic action of GH and insulin on IGF-I and -II expression in carp hepatocytes. **(A)** Time course of GH alone (300 ng/ml), insulin alone (10 nM) and co-treatment with GH (300 ng/ml) and insulin (10 nM) on IGF-I and -II mRNA expression. **(B)** Dose dependence of increasing levels of insulin (0.01–100 nM) on IGF-I and -II mRNA expression induced by GH treatment (300 ng/ml). **(C)** As a reciprocal experiment, increasing levels of GH treatment (10–1000 ng/ml) on IGF-I and -II mRNA expression induced by insulin (10 nM) was also tested in carp hepatocytes. In dose-response studies, the duration of drug treatment was fixed at 24 h. In these experiments, total RNA was isolated from hepatocytes after drug treatment and used for real-time PCR for IGF-I and -II mRNA measurement. IGF data for individual groups denoted by different letters represent a significant difference at *P* < 0.05.

Since both GH and insulin were capable of inducing IGF expression in serum-free culture of carp hepatocytes, co-treatment experiments were also performed to test the possible additivity/potentiation on IGF-I and -II responses induced by the two stimulants. In our time course study, GH (300 ng/ml) and insulin (10 nM) alone were both effective in elevating IGF-I and -II mRNA levels in a time-dependent manner with significant rises observed at 24 h (Figure 3A). GH and insulin alone did not alter IGF expression after 12 h incubation, but significant rises in IGI-I and -II mRNA levels could be noted with co-treatment of the two stimulants. With the treatment extended to 24 h, the potentiating effects on IGF-I and -II gene expression were markedly elevated by GH and insulin co-treatment (~900% Ctrl), which were 3–4-fold higher than the corresponding responses induced by GH (~200% Ctrl) and insulin alone (~300%). To test for the dose-dependence of the potentiating effects, hepatocytes were challenged with GH (300 ng/ml) for 24 h in the presence of increasing levels of insulin (0.01–100 nM). In this case, GH-induced IGF-I and-II mRNA expression was noticeably enhanced in a dose-related fashion by insulin, especially in the 0.1–100 nM dose range (Figure 3B). Similar results were also observed in reciprocal experiment with hepatocytes exposed to insulin (10 nM) with co-treatment of increasing concentrations of GH (10–1000 ng/ml) and parallel enhancement of insulin-induced IGF-I and -II gene expression by GH could be observed in the 30−1000 ng/ml dose range (Figure 3C).

**Figure 4:**
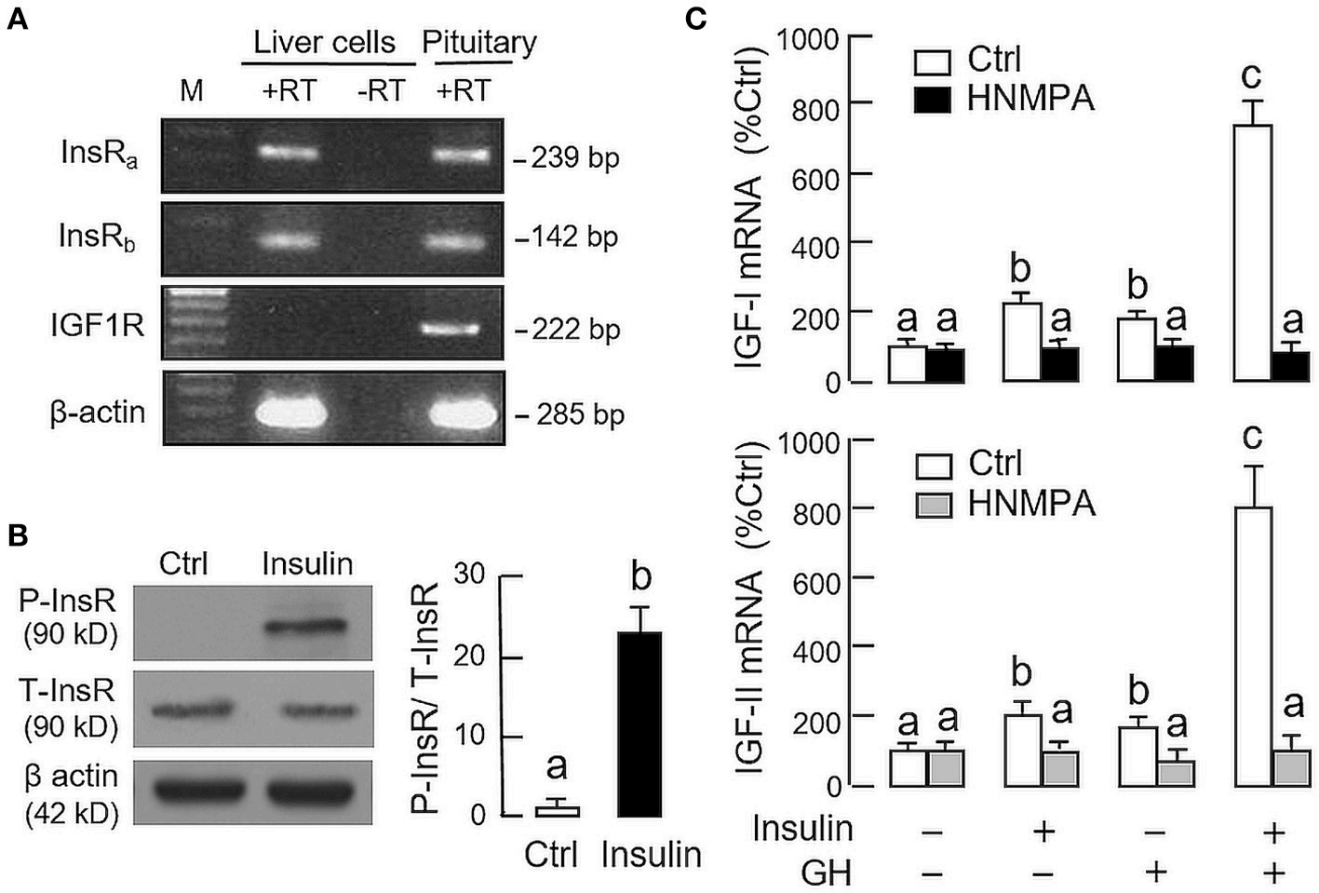
Receptor specificity for insulin potentiation of GH-induced IGF-I and -II expression in carp hepatocytes. **(A)** RT-PCR for InsR_a_, InsR_b_, and IGF1R expression in carp hepatocytes. PCR with primers for respective gene targets was performed in total RNA isolated from carp hepatocytes with (+RT) or without reverse transcription (-RT). Parallel PCR for the same targets in total RNA prepared from the carp pituitary (with reverse transcription) was used as positive control while PCR for β actin was used as the internal control. (M: Size markers for PCR products) **(B)** Insulin treatment on protein phosphorylation of InsR in carp hepatocytes. Cell lysate was prepared from hepatocytes after 15-min treatment with insulin (10 nM) and used in Western blot with antibodies for the phosphorylate form (P-form) and total protein (T-form) of InsR. The signals for the two forms of InsR were quantitated by Image J and expressed as a ratio of P-form/T-form in the bar graphs on the right. In this experiment, parallel blotting with α actin was used as the loading control. **(C)** Blockade of InsR activation on insulin potentiation of GH-induced IGF-I and -II mRNA expression in carp hepatocytes. Hepatocytes were incubated for 24 h with GH alone (300 ng/ml), insulin alone (10 nM) or co-treatment of GH (300 ng/ml) and insulin (10 nM) in the presence of HNMPA (10 μM), an inhibitor for InsR activation. After that, total RNA was isolated and used for real-time PCR measurement of IGF-I and -II mRNA. For the data presented, the groups denoted by different letters represent a significant difference at *P* < 0.05.

Given that insulin and IGF-I are structurally related and known to have cross-reactivity at receptor level (32), the receptor specificity for the potentiating effect of insulin on GH-induced IGF expression was also examined. As revealed by RT-PCR using primers specific for grass carp InsR and IGF1R, PCR signals for two isoforms of InsR, namely InsRa and InsRb, but not IGF1R could be noted in hepatocytes prepared from grass carp (Figure 4A). The lack of IGF1R signal caused by RNA decay during sample preparation is rather unlikely as the corresponding signals for β actin (the internal control) were consistently detected in the samples examined. To test if human insulin indeed can trigger InsR activation in the carp liver, carp hepatocytes were challenged with insulin (10 nM) for 15 min, and in this case, a rapid phosphorylation in InsR was observed after drug treatment (Figure 4B). To further confirm the role of InsR activation in the potentiating effects of insulin on GH-induced IGF-I and -II expression, carp hepatocytes were treated for 24 h with GH (300 ng/ml) and insulin (10 nM), alone or in combination, in the presence of HNMPA (10 μM), a blocker for InsR activation. As shown in Figure 4C, both the stimulatory effect of insulin on basal as well as its potentiating effects on GH-induced IGF-I and -II mRNA expression were abolished by HNMPA treatment. Interestingly, HNMPA was also effective in blocking the stimulatory effects of GH on IGF-I and -II gene expression. In parallel experiment, however, treatment with the IGF1R inactivator PPP (10 μM) did not have significant effects on insulin potentiation of IGF-I and -II responses induced by GH (746.8 ± 95.4%Ctrl *vs*. 820.4 ± 88.7%Ctrl for IGF-I mRNA and 717.6 ± 90.8%Ctrl *vs*. 792.2 ± 107.8%Ctrl for IGF-II mRNA for the groups with GH and insulin co-treatment in the presence or absence of PPP, *P* > 0.05).

### Mechanisms for GH and insulin synergism on IGF-I and -II expression

**Figure 5:**
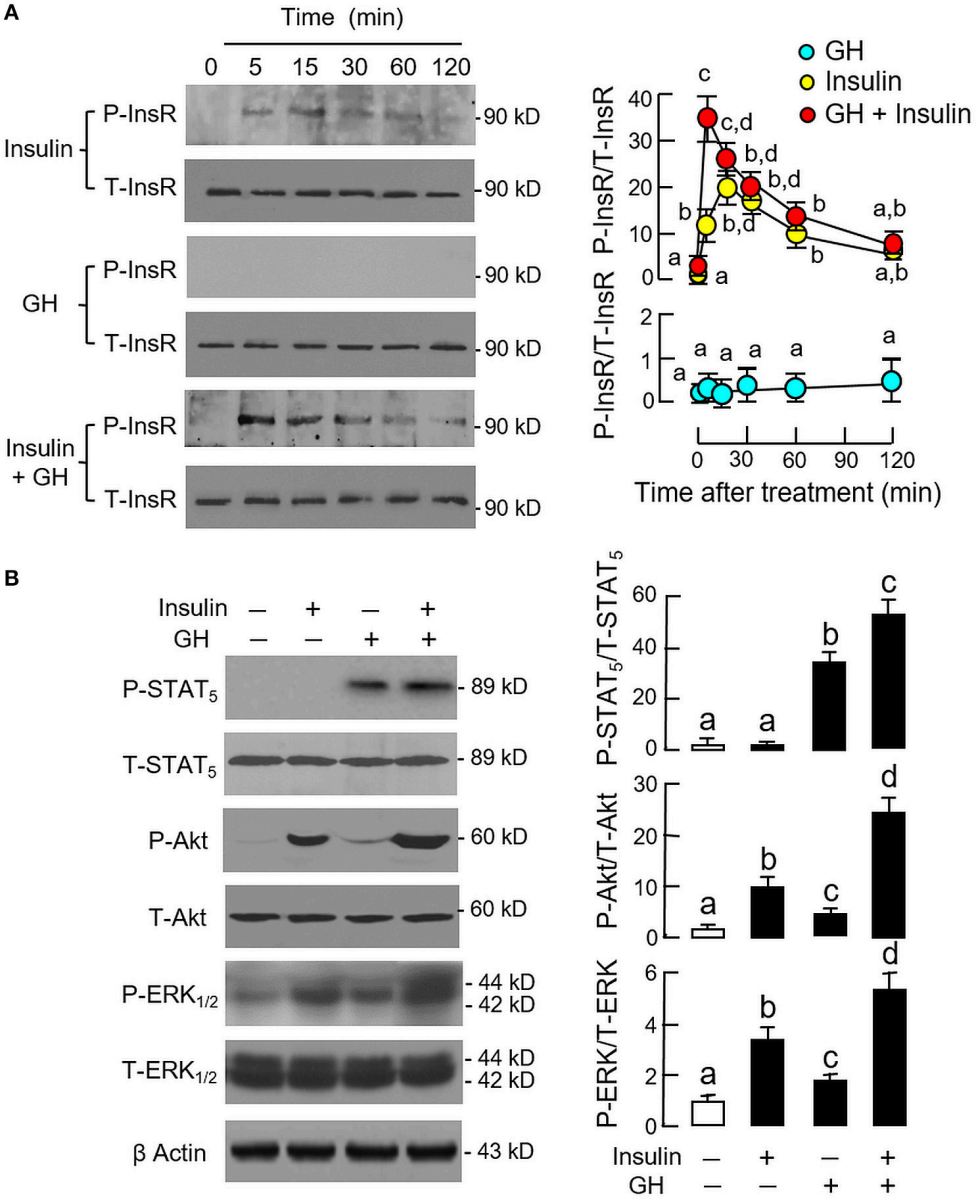
Synergistic effect of GH and insulin on InsR, STAT_5_, Akt, and ERK_1/2_ activation in carp hepatocytes. **(A)** GH enhancement of InsR phosphorylation induced by insulin treatment. **(B)** Insulin potentiation of STAT_5_, Akt, and ERK_1/2_ phosphorylation induced by GH treatment. In these experiments, hepatocytes were exposed to insulin alone (10 nM), GH alone (300 ng/ml) or co-treatment of GH (300 ng/ml) and insulin (10 nM) for the duration as indicated for InsR phosphorylation. For STAT_5_, Akt, and ERK_1/2_ phosphorylation, the duration of drug treatment was reduced to 15 min. After treatment, cell lysate was prepared and used for Western blot with antibodies for phosphorylated form (P-form) and total protein (T-form) of the respective targets. The signals for the two forms of target proteins were then quantitated by Image J and expressed as a ratio of P-form/T-form in the bar graphs on the right. In these experiments, parallel blotting of β actin was also conducted to serve as the loading control.

Since InsR activation was found to be essential for the potentiating effect of insulin, the effect of co-treatment with GH and insulin on InsR activation was also examined in carp hepatocytes (Figure 5A). In this case, insulin (10 nM) but not GH (300 ng/ml) was effective in inducing a time-dependent phosphorylation of InsR with peak response observed at 15 min after drug treatment. Phosphorylation of InsR was further enhanced by co-treatment with GH (300 ng/ml) and insulin (10 nM) with the peak response shifted to 5 min. Of note, GH and insulin alone or in combination up to 120 min did not have noticeable effect on total protein of InsR expressed in hepatocytes. In parallel experiment, a 15-min treatment with GH (300 ng/ml) but not insulin (10 nM) was effective in triggering a rapid phosphorylation of STAT_5_ in carp hepatocytes and this stimulatory effect could be further enhanced with GH and insulin co-treatment (Figure 5B). In the same study, GH and insulin alone could also induce Akt and ERK_1/2_ phosphorylation and these stimulatory effects were notably intensified by GH and insulin co-treatment.

**Figure 6:**
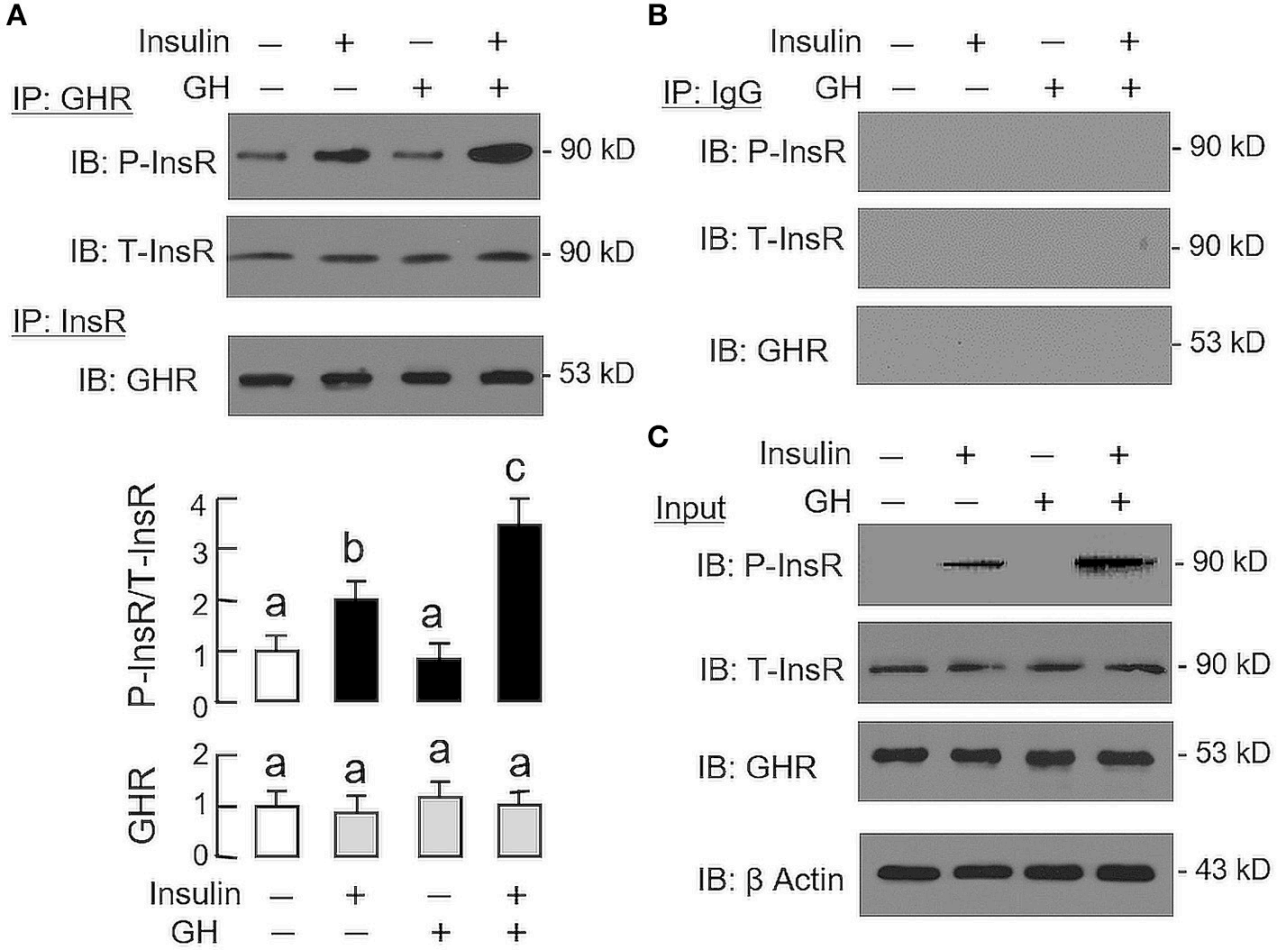
Protein:protein interaction of GHR and InsR expressed in carp hepatocytes. Membrane protein extract was prepared from hepatocyte after challenged for 15-min with insulin alone (10 nM), GH alone (300 ng/ml) or co-treatment with GH (300 ng/ml) and insulin (10 nM) and used in immunoprecipitation (IP) with antibody for GHR or total protein of InsR **(A)**. For protein samples pulled down by IP against GHR, size-fractionation by SDS-PAGE was conducted followed by immunoblotting (IB) with antibodies for phosphorylated form (P-InsR) and total protein of InsR (T-InsR). For protein samples pulled down by IP against total protein of InsR, similar SDS-PAGE coupled with IB using the antibody for GHR was also performed. The IB signals for the two forms of InsR and GHR were quantitated by Image J and presented in the bar graphs below the IB results. In this study, IP with mouse IgG followed by IB for the respective targets was used as the negative control **(B)**. Parallel IB using the protein extract prior to IP was used as the input control while the corresponding blotting for β actin in whole cell lysate was also conducted to serve as the loading control **(C)**.

Given that heterodimerization of membrane receptors on cell surface is known to modify post-receptor signaling coupled to the respective receptors (33), the possible association of GHR with InsR in carp hepatocytes was also tested in plasma membrane by co-immunoprecipitation (Figure 6). In membrane protein samples prepared from hepatocytes after 15-min treatment with GH alone (300 ng/ml), insulin alone (10 nM), or a combination of both (as input), except for a raise in immunoblotting (IB) signal for phosphorylated InsR with insulin alone as well as its notable potentiation by GH co-treatment, the IB signals for total InsR and GHR were not altered by insulin and GH, alone or in combination (Figure 6C). After protein pull-down by the antiserum for GHR (as IP by GHR), the IB signal for phosphorylated InsR could be observed in the control group and the signal intensity was not altered by GH treatment. However, the corresponding signal could be up-regulated by insulin with notable enhancement by GH co-treatment. In the same study, the IB signal for total InsR was not modified by insulin or GH or a combination of both (Figure 6A). In reciprocal experiment with pull-down by the antibody for total InsR (as IP by InsR), the IB signal for GHR was also not affected by similar drug treatment. The signals based on IP with GHR and InsR antibodies were confirmed to be specific, as similar IB signals were not detected with parallel IP using mouse IgG (as negative control for the experiment, Figure 6B).

**Figure 7:**
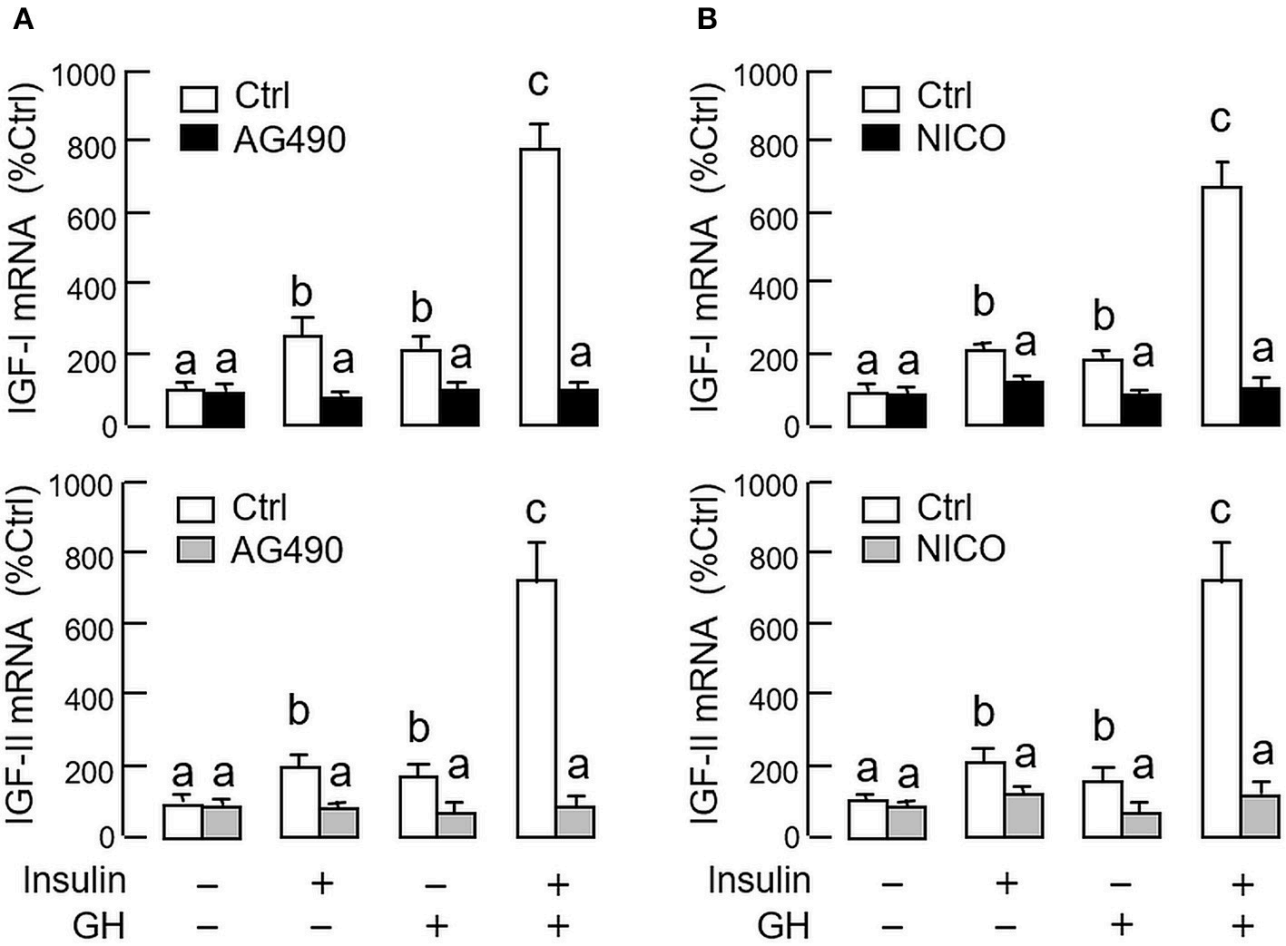
Functional role of JAK_2_/STAT_5_ pathway in the synergistic effect of GH and insulin on IGF-I and -II expression in carp hepatocytes. **(A)** JAK_2_ blockade or **(B)** STAT_5_ inactivation on insulin potentiation of GH-induced IGF-I and -II mRNA expression at the hepatic level. In this study, hepatocytes were challenged for 24 h with insulin alone (10 nM), GH alone (300 ng/ml) or co-treatment with GH (300 ng/ml) and insulin (10 nM) in the presence of the JAK_2_ inhibitor AG490 (20 μM) or STAT_5_ inactivator NICO (20 μM). After treatment, total RNA was isolated and used for real-time PCR for IGF-I and -II mRNA. For IGF data presented, the groups denoted by different letters represent a significant difference at *P* < 0.05.

**Figure 8:**
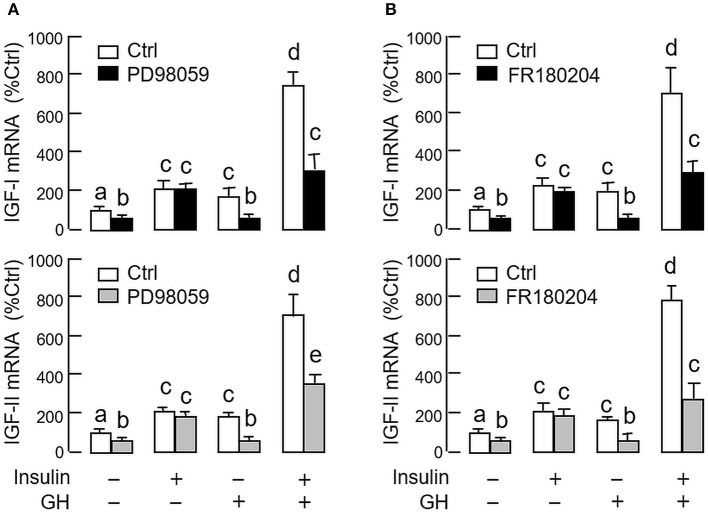
Functional role of MEK_1/2_/ERK_1/2_ pathway in the synergistic effect of GH and insulin on IGF-I and -II expression in carp hepatocytes. **(A)** MEK_1/2_ blockade or **(B)** ERK_1/2_ inhibition on insulin potentiation of GH-induced IGF-I and -II mRNA expression at the hepatic level. In this study, hepatocytes were challenged for 24 h with insulin alone (10 nM), GH alone (300 ng/ml) or co-treatment of GH (300 ng/ml) and insulin (10 nM) in the presence of the MEK_1/2_ inhibitor PD98059 (10 μM) or ERK_1/2_ inhibitor FR180204 (2 μM). After that, total RNA was isolated and used for real-time PCR for IGF-I and -II mRNA. For IGF data presented, the groups denoted by different letters represent a significant difference at *P* < 0.05.

**Figure 9:**
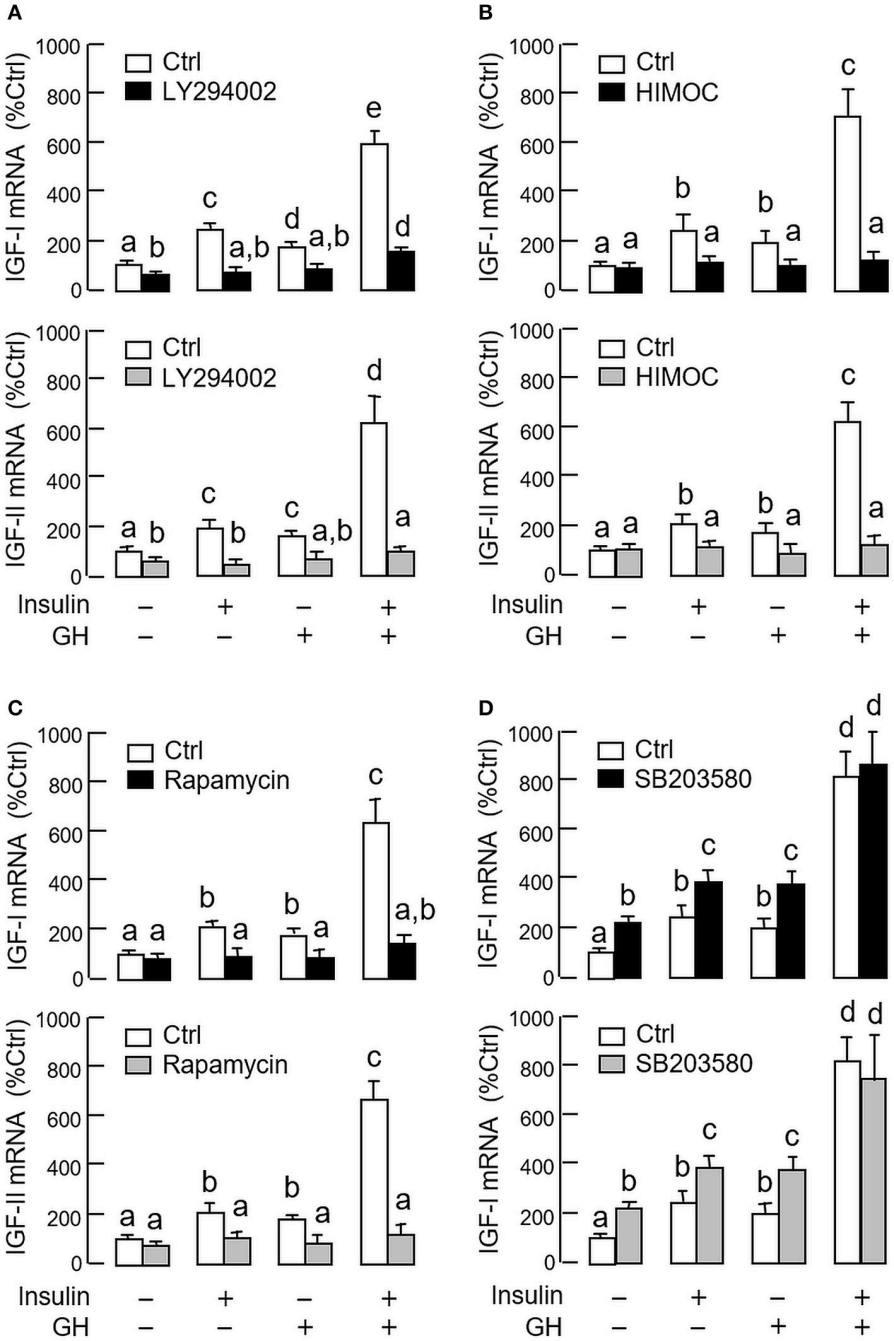
Functional role of P38 MAPK and PI3K/Akt pathways in the synergistic effect of GH and insulin on IGF-I and -II expression in carp hepatocytes. Effects of inhibiting **(A)** PI3K, **(B)** Akt, **(C)** mTOR and **(D)** P38 MAPK on insulin potentiation of GH-induced IGF-I and -II mRNA expression at the hepatic level. Hepatocytes were incubated for 24 h with insulin alone (10 nM), GH alone (300 ng/ml) or co-treatment with GH (300 ng/ml) and insulin (10 nM) in the presence of the PI3K inhibitor LY294002 (10 μM), Akt inhibitor HIMOC (10 μM), mTOR inhibitor rapamycin (20 nM) and P38 MAPK inhibitor SB203580 (5 μM), respectively. After that, total RNA was isolated and used for real-time PCR for IGF-I and -II mRNA. For IGF data presented, the groups denoted by different letters represent a significant difference at *P* < 0.05.

As revealed by the results of Western blot, co-treatment with GH and insulin could enhance InsR activation with parallel aggravation of STAT_5_, ERK_1/2_ and Akt phosphorylation at the hepatic level. The possible involvement of JAK/STAT, MAPK and PI3K/Akt cascades in the synergistic effect of GH and insulin on IGF-I and -II regulation was also investigated using a pharmacological approach. In carp hepatocytes, co-treatment with GH (300 ng/ml) and insulin (10 nM) for 24 h consistently induced a notable potentiation of IGF-I and -II mRNA expression when compared to the corresponding responses caused by GH or insulin alone (Figures 7–9). Except for the P38 MAPK inhibitor SB203580 (5 μM, Figure 9D), the synergistic actions of GH and insulin on IGF-I and -II mRNA expression were suppressed/totally negated by simultaneous treatment with the JAK_2_ inhibitor AG490 (20 μM, Figure 7A), STAT_5_ inactivator NICO (20 μM, Figure 7B), MEK_1/2_ blocker PD98059 (10 μM, Figure 8A), ERK_1/2_ inhibitor FR180204 (2 μM, Figure 8B), PI3K inactivator LY294004 (10 μM, Figure 9A), Akt inhibitor HIMOC (10 μM, Figure 9B) or mTOR blocker rapamycin (20 nM, Figure 9C). Treatment with the P38 MAPK inhibitor SB203580 alone was found to elevate basal levels of IGF-I and -II mRNA and notably enhance the corresponding responses induced by GH or insulin alone (Figure 9D). Interestingly, SB203580 did not alter the synergistic effects on the two IGFs induced by GH and insulin co-treatment. Similar to our preceding studies, transcript expression of IGF-I/-II induced by GH or insulin alone could be blocked by inhibiting JAK_2_/STAT_5_ pathway with AG490 and NICO (Figure 7) or inactivation of PI3K/Akt pathway with LY294004, HIMOC and rapamycin (Figure 9). Although inactivation of MEK/ERK cascade using PD98059 and FR180204 was shown to suppress GH-induced IGF-I and -II mRNA expression, similar treatment was not effective in blocking the corresponding responses induced by insulin (Figure 8).

## Discussion

The somatotropic axis with GH-induced IGF-I synthesis and secretion in the liver is well conserved from fish to mammals (34). In fish species, e.g., salmon (8) and tilapia (7), GH is also effective in triggering IGF-II expression at the hepatic level. This is different from mammals in which IGF-II expression is not responsive to GH, and in general, not considered as a part of the somatotropic axis. At present, the signal transduction for GH-induced IGF-II expression is still unknown and further studies are clearly warranted in fish models. In our recent study, GH was shown to induce rapid phosphorylation of JAK_2_, STAT_5_, MEK_1/2_, ERK_1/2_, P38 MAPK, and Akt in grass carp hepatocytes (27), suggesting that the JAK_2_/STAT_5_, MAPK and PI3K/Akt pathways may play a role in hepatic functions of GH in carp species. This idea is supported by the current study with the same cell model under a serum-free culture condition, in which GH-induced IGF-I and -II mRNA expression could be negated by (i) inhibiting JAK_2_ and STAT_5_ (by AG490 and NICO, respectively), (ii) blocking MEK_1/2_ and ERK_1/2_ (by PD98059 and FR180204, respectively), and (iii) inactivating PI3K and Akt (by LY294004 and HIMOC, respectively). Besides, inhibition of mTOR (by rapamycin), the downstream effector of Akt, could also nullify the effects of GH, but similar blockade of P38 MAPK (by SB203580), another component of MAPK signaling, was not effective in this regard. These results may imply that GH can stimulate IGF-I and -II gene expression in the carp liver via activation of the JAK_2_/STAT_5_, MEK_1/2_/ERK_1/2_ and PI3K/Akt/mTOR pathways. Apparently, P38 MAPK of the MAPK cascades is not involved in the IGF responses induced by GH. Our results are highly comparable to the previous report in trout hepatocytes with GH-induced IGF-I expression mediated by the JAK_2_/STAT_5_, ERK_1/2_ and PI3K/Akt signaling (30). To our knowledge, our study also represents the first report to elucidate the post-receptor signal transduction for GH-induced IGF-II expression in fish species.

In mammals, hepatic functions (e.g., glucose and lipid metabolism) are known to be under the regulation of pancreatic hormones, mainly though the connection via the portal vascular link between pancreatic islets and the liver (12). The vascular link also allows for a functional cross-talk between nutritional status and somatotropic axis. For examples, insulin, an endocrine signal commonly observed during positive energy balance, is capable of stimulating IGF-I release (35) and transcript expression in rat hepatocytes (22). In hepatocytes prepared from rat fetus, similar treatment with insulin can also elevate IGF-II mRNA levels with parallel enhancement in IGF-II transcript stability (20). For the direct effect of insulin acting at the hepatic level in fish models, insulin had been reported to reduce IGF-I gene expression in tilapia hepatocytes (7) but with no effect in similar study in salmon hepatocytes (26). In both cases, hepatic expression of IGF-II mRNA could be up-regulated by insulin (7, 8) but the mechanisms involved are still unclear. In our current study with carp hepatocytes, insulin treatment not only could trigger IGF-I and -II mRNA expression but also induce rapid phosphorylation of MEK_1/2_, ERK_1/2_, P38 MAPK, and Akt. In parallel experiments, insulin-induced IGF-I and -II mRNA expression were also negated by inactivation of PI3K, Akt and mTOR (by LY294004, HIMOC and rapamycin, respectively) but similar blockade of MEK_1/2_, ERK_1/2_, and P38 MAPK (by PD98059, FR180204 and SB203580, respectively) were not effective in these regards. Although the biological actions of insulin are known to be mediated by the MAPK and PI3K/Akt cascades (31), our results suggest that insulin can induce IGF-I and -II gene expression in the carp liver via activation of the PI3K/Akt/mTOR pathway whereas the MAPK cascades may be involved in hepatic functions of insulin other than IGF regulation. In carp hepatocytes, insulin did not trigger JAK_2_ and STAT_5_ phosphorylation but the elevation in IGF-I and -II mRNA levels induced by insulin were blocked by inhibiting JAK_2_ and STAT_5_ (by AG490 and NICO, respectively). Our data clearly indicate that the pathway is not activated by insulin in the carp liver, and interestingly, the IGF responses induced by insulin are dependent on the normal functioning of the JAK_2_/STAT_5_ cascade. Similar to the preceding study with GH as the stimulant, inhibiting P38 MAPK (by SB203580) was not effective in blocking insulin induction on IGF-I and -II mRNA expression. In contrast, P38 MAPK inhibition was found to increase basal as well as GH- and insulin-induced IGF-I and -II responses, implying that P38 MAPK signaling may have negative effect on IGF expression at the hepatic level. These findings raise the possibility that P38 MAPK activation induced by GH and insulin may play a role in signal termination for the stimulatory responses of IGF-I and -II. Of note, the synergistic effect of GH and insulin was not further enhanced by P38 MAPK inhibition. Apparently, the inhibitory action of P38 MAPK in the carp liver could be noted with GH or insulin alone but not with the co-treatment of the two stimulants.

Although functional interaction of insulin and GH has been well-documented in mammals, the effects are highly variable and dependent on the duration of drug treatment (17, 23), tissue concerned (36, 37), nutritional states/energy balance (15) and functional status of GH (38) and insulin (14). In rat hepatocytes, short-term treatment of insulin has been shown to have either no effect (21) or potentiation of GH-induced IGF-I expression (22), while prolonged treatment with insulin can inhibit the hepatic actions of GH by down-regulation of GHR expression in the liver (18). At present, except for a single report in chicken (25) and two reports in fish models, including salmon (26) and tilapia (7), the information is rather limited in non-mammalian species for IGF-I regulation by functional interaction of GH and insulin at hepatocyte level. In the study for chicken hepatocytes, a potentiating effect on IGF-I expression by co-treatment with GH and insulin has been reported (25). However, the corresponding effects in fish models are more variable. In tilapia hepatocytes, insulin can reduce basal but with no effect on GH-induced IGF-I gene expression (7). In salmon hepatocytes, interestingly, insulin has no effect on basal but inhibits GH-induced IGF-I mRNA expression (26). In both cases, insulin can consistently enhance the stimulatory effect of GH on IGF-II gene expression at hepatocyte level (7, 8). In our study with carp hepatocytes, co-treatment with GH and insulin was found to induce a notable potentiation in IGF-I and -II mRNA expression in a time- and dose-related fashion. Although insulin and IGF-I are known to have cross-reactivity at receptor level (32) and InsR and IGF1R have also been reported to form hybrid receptor (39), only the transcript signals for InsR but not IGF1R could be detected in carp hepatocytes. In the same cell model, insulin was effective in triggering rapid phosphorylation of InsR and the potentiating effects of insulin on GH-induced IGF-I and -II mRNA expression could be blocked by inactivating InsR (by HNMPA) but not IGF1R (by PPP). These findings indicate that insulin can interact with GH in a synergistic manner to up-regulate IGF-I and -II gene expression in the carp liver via InsR activation. These results further support our idea for IGF regulation by functional interaction of GH and insulin in fish models (see introduction). At the hepatic level, the effect of insulin on IGF-I regulation by GH is species-specific (e.g., with stimulation in grass carp, inhibition in salmon and no effect in talipia) whereas the potentiating/enhancing effect of insulin on the corresponding responses for IGF-II appears to be a common phenomenon in fish species.

Regarding the mechanisms underlying the synergistic actions of GH and insulin on IGF-I and -II expression, our co-immunoprecipitation study with membrane proteins prepared from carp hepatocytes has shown that InsR could be physically associated with GHR under basal condition. Upon insulin stimulation, InsR phosphorylation (indicative of InsR activation) was initiated at hepatocyte level and this stimulatory action could be further aggravated with GH co-treatment. Meanwhile, a noticeable rise in GHR association with phosphorylated InsR was observed with parallel enhancement in the phosphorylation of STAT_5_ (compared to GH alone) and ERK_1/2_ and Akt (compared to GH or insulin alone). Consistent with these findings, the synergistic effect of GH and insulin on IGF-I and -II mRNA expression could be reduced/totally abolished in carp hepatocytes by (i) inhibiting JAK_2_ and STAT_5_ (by AG490 and NICO, respectively), (ii) blocking MEK_1/2_ and ERK_1/2_ (by PD98059 and FR180204, respectively) or (iii) inactivating PI3K, Akt and mTOR (by LY294002, HIMOC and rapamycin, respectively). Although heterodimerization of membrane receptors (e.g., G protein-coupled receptors) has been reported in plasma membrane of mammalian cells and plays a role in modification of post-receptor signaling and downstream functions (33), “dimerization” between GHR and InsR with subsequent enhancement in InsR activation has not been documented previously. Based on our findings, we postulate that protein: protein interaction of GHR and InsR may have a functional role in the synergistic action of GH and insulin on IGF regulation in the carp liver. Presumably, GH activation of GHR with protein: protein interaction with InsR not only can enhance InsR activation by insulin at membrane level, but also intensify the signal transduction via STAT_5_-, ERK_1/2_- and Akt-dependent mechanisms. The subsequent enhancement in transcriptional events associated with the JAK_2_/STAT_5_, MEK_1/2_/ERK_1/2_, and PI3K/Akt/mTOR pathways may then contribute to the potentiating effect observed in IGF-I and -II mRNA expression in carp hepatocytes.

In mammals, the effects of insulin on hepatic actions induced by GH is highly dependent on the duration of drug treatment (23). Based on the study in H4IIE hepatoma, short-term treatment with insulin (up to hours) can enhance GH-induced MEK_1/2_ and ERK_1/2_ phosphorylation. During the process, modification of Shc/GRB_2_/SOS complex formation with facilitation of membrane translocation of MEK_1/2_ can also be noted, although their functional relevance in IGF regulation has yet to be determined (24). Prolonged treatment with insulin (>10 h), on the contrary, is known to suppress GH signaling at both receptor and post-receptor levels. At the receptor level, e.g., in HuH7 and H4 cells, continuous treatment with insulin for long period not only can reduce membrane translocation of GHR (36) but also down-regulate GHR protein synthesis (16). These inhibitory effects also occur with parallel drop in GHR mRNA and gene transcription mediated by insulin activation of PI3K and MEK_1/2_/ERK_1/2_ (18, 19). At the post-receptor level, prolonged treatment with insulin is known to inhibit JAK_2_/STAT_s_ signaling by reducing JAK_2_ and STAT_5_ phosphorylation (16) and protein content of STAT_3_, e.g., in H4IIE hepatoma cells (17). In other cell lines, e.g., human HeG2 and rat hepatoma cells, expression of SOCS, the intracellular signal terminators for GH (40), can also inhibit InsR activation and its post-receptor signaling via IRS, Akt and ERK_1/2_ (41), which may add another facet on the functional interaction between GH and insulin. Apparently, the synergistic action of GH and insulin in carp hepatocytes is similar and yet distinct from that in mammals. In both cases, potentiation at the level of post-receptor signaling can be noted as reflected by the enhancement in protein phosphorylation of various kinases involved in signal transduction. In fish models, insulin can interact with GH to regulate the anabolic and catabolic functions mediated by GHR at the hepatic level (42). In trout hepatocytes, the effects of GH on lipolysis by regulating the expression of hormone sensitive lipase can be modified by insulin co-treatment. During the process, insulin is also effective in elevating the levels of JAK_2_ and STAT_5_ phosphorylation induced by GH (43), indicating that GH and insulin interaction on post-receptor signaling is not restricted to IGF regulation in the liver of fish species. In grass carp, the potentiating effects on IGF-I and -II expression can be noted only after 12–24 h drug treatment. This is at variant with the inhibitory action associated with prolonged treatment of insulin in mammals and may suggest that the functional interaction of GH and insulin in the carp liver may have a late onset for IGF responses with resistance to GHR down-regulation and/or signal termination in post-receptor signaling.

**Figure 10:**
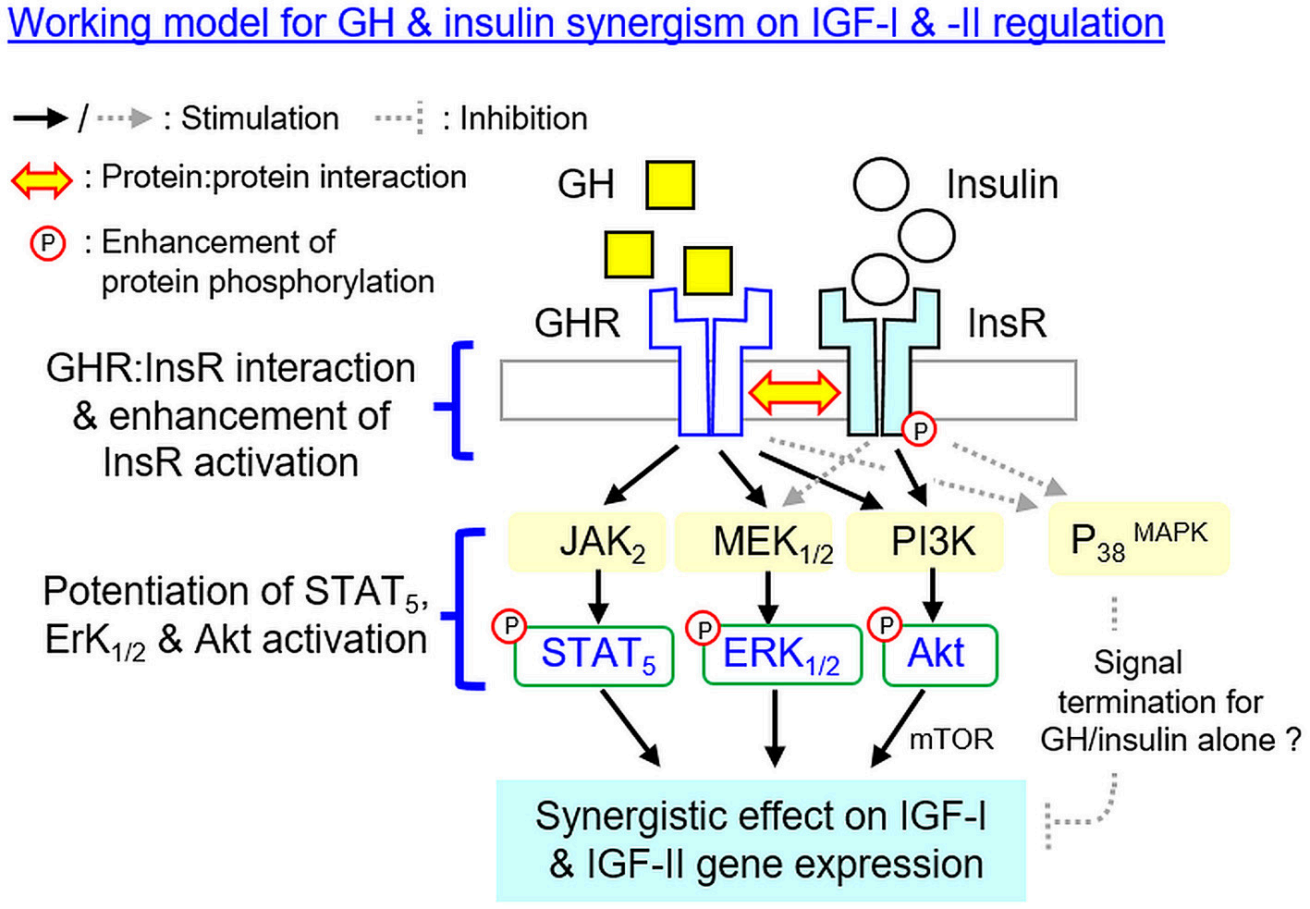
Working model proposed for the synergistic action of GH and insulin on IGF-I and -II expression in the carp liver. In carp hepatocytes, IGF-I and -II mRNA expression can be up-regulated by GH via the JAK_2_/STAT_5_, MEK_1/2_/ERK_1/2_, and PI3K/Akt/mTOR cascades. Insulin also has similar effects on IGF-I and -II gene expression but these actions are mediated via PI3K/Akt/mTOR but not JAK_2_/STAT_5_ and MEK_1/2_/ERK_1/2_ pathways. At the hepatic level, IGF-I and -II responses induced by GH can be markedly enhanced with insulin co-treatment. This potentiating effect can be observed with protein:protein interaction of GHR and InsR at the membrane level together with notable enhancement in InsR phosphorylation. Meanwhile, the levels of STAT_5_, ERK_1/2_, and Akt phosphorylation can also be potentiated by GH and insulin co-treatment. The aggravation in JAK_2_/STAT_5_, MEK_1/2_/ERK_1/2_ and PI3K/Akt signaling caused by simultaneous activation of GHR and InsR probably can contribute to the synergist effect on IGF-I and -II expression. Of note, MEK_1/2_ and ERK_1/2_ activation can also be induced by insulin but the pathway is not involved in insulin-induced IGF-I and -II expression. However, the functional role of MEK_1/2_/ERK_1/2_ activation by insulin in the potentiating effects caused by GH and insulin co-treatment should not be excluded. In carp model, P38 MAPK of MAPK cascades is not involved in the synergistic action of GH and insulin. P38 MAPK may play a role in signal termination for the IGF responses induced by GH or insulin respectively, and interestingly, its inhibitory effect can be nullified by co-treatment with GH and insulin but the mechanisms involved have yet to be elucidated.

In summary, using grass carp hepatocytes as a model, we have elucidated the post-receptor signaling mechanisms for IGF-I and -II gene expression in the carp liver induced by GH and insulin separately as well as by co-treatment of the two together (Figure 10). In grass carp, GH could up-regulate IGF-I and -II mRNA expression at hepatocyte level by functional coupling with the JAK_2_/STAT_5_, MEK_1/2_/ERK_1/2_ and PI3K/Akt/mTOR pathways. Similar stimulation on transcript expression of the two IGFs was also noted with insulin treatment but the effects were mediated by PI3K/Akt/mTOR but not JAK_2_/STAT_5_ and MEK_1/2_/ERK_1/2_ cascades. With GH and insulin co-treatment, a notable enhancement of IGF-I and -II mRNA expression was observed in carp hepatocytes and these potentiating effects presumably were mediated by protein:protein interaction of GHR and InsR at the membrane level followed by potentiation of post-receptor signailng coupled with the two receptors. Apparently, GHR activation by GH could promote InsR phosphorylation induced by insulin via protein:protein interactions of the two receptors, which might then aggravate protein phosphorylation of STAT_5_, ERK_1/2_ and Akt at hepatocyte level. The subsequent enhancement in JAK_2_/STAT_5_, MEK_1/2_/ERK_1/2_, and PI3K/Akt signaling probably could lead to the synergistic effect on IGF-I and -II mRNA expression observed with GH and insulin co-treatment. Of note, MEK_1/2_ and ERK_1/2_ activation can also be induced by insulin but the pathway is not involved in insulin-induced IGF-I/-II expression. Nevertheless, the functional role of MEK_1/2_/ERK_1/2_ activation by insulin in the potentiating effects caused by GH and insulin co-treatment should not be excluded. In our study, the P38 MAPK component of MAPK, despite its activation by GH and insulin, did not appear to be involved in the synergistic action for IGF expression. In the carp liver, P38 MAPK might act as a part of the signal termination for IGF-I and -II responses induced by GH or insulin alone, and interestingly, these inhibitory effects could be nullified by GH and insulin co-treatment but the mechanism involved is still unclear. Our study, as a whole, provides new information on the mechanistic aspects for IGF regulation by hepatic interaction of GH and insulin in a carp species, which will also enhance our understanding on the signal crosstalk between energy homeostasis and somatotropic axis. Based on the previous studies in human and rodents, functional interaction of GH and insulin has been reported in glucose homeostasis (13, 38), lipid metabolism (13, 13), GHR regulation (36, 37) and insulin signaling (23, 41). It will be of interest to extend our study on GHR and InsR interaction to mammalian species to test if it is a common phenomenon in vertebrate evolution.

## Data availability

The raw data supporting the conclusions of this manuscript will be made available by the authors, without undue reservation, to any qualified researcher.

## Author contributions

AW was the PI and grant holder. AW and QJ were responsible for project planning and data analysis. QJ and JB were involved in functional studies with carp hepatocytes while MH was responsible for protein phosphorylation experiments. Manuscript preparation was done by AW and KY.

### Conflict of interest statement

The authors declare that the research was conducted in the absence of any commercial or financial relationships that could be construed as a potential conflict of interest.

## Abbreviations

GH: Growth hormone
IGF: Insulin-like growth factor
GHR: GH receptor
InsR: Insulin receptor
IGF1R: IGF-I receptor
MAPK: Mitogen-activated protein kinases
MEK_1/2_: Mitogen-activated protein kinase kinase 1/2
ERK_1/2_: Extracellular signal-regulated kinase 1/2
P38 MAPK: P38 Mitogen-activated protein kinase
PI3K: Phosphoinositide 3-kinase
Akt: Protein kinase B
mTOR: Mechanistic target of rapamycin
JAK_2_: Janus kinase 2
STAT_5_: Signal transducer and activator of transcription 5
