# Supplementary material for: Mechanisms Underlying the Synergistic Action of Insulin and Growth Hormone on IGF-I and -II Expression in Grass Carp Hepatocytes
Source: Front Endocrinol (Lausanne). 2018 Jun 21;9:336. doi: 10.3389/fendo.2018.00336 (PMC6021495; doi:10.3389/fendo.2018.00336)
Supplement: Supplementary file 1 [file Image_1.PDF]

Supplemental Fig.1

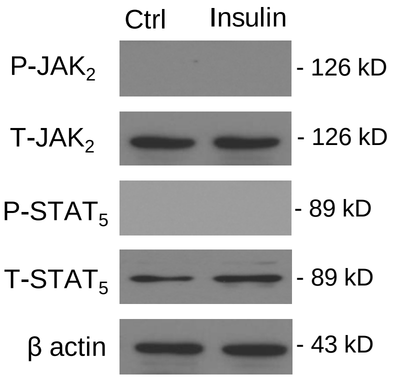

Suppl Fig.1 Effects of insulin on JAK_2 and $STAT_5$ phosphorylation in grass carp hepatocytes. Cell lysate was prepared from hepatocytes after 15-min treatment with insulin (10 nM) and subjected to Western bloc using specific antibodies for the phosphorylated form (P-form) and total protein (T-form) of JAK_2 and $STAT_5$, respectively. Parallel blotting of β actin was used as the loading control.
